# The risk of anaphylaxis on holidays

**DOI:** 10.1097/ACI.0000000000001014

**Published:** 2024-08-02

**Authors:** Erminia Ridolo, Alessandro Barone, Martina Ottoni, Francesca Nicoletta

**Affiliations:** Allergy and Clinical Immunology, Medicine and Surgery Department, University of Parma, Parma, Italy

**Keywords:** allergy, anaphylaxis, food, holiday, hymenoptera

## Abstract

**Purpose of review:**

The goal of this review is to summarize the potential causes of anaphylaxis in the different holiday contexts, providing practical suggestions aimed to mitigate the stress challenged by allergic patients because of unfamiliar situations.

**Recent findings:**

A regard was reserved to potential food triggers, particularly uncommon ones and typical of certain destinations, and to arthropods responsible for anaphylaxis.

**Summary:**

This review highlights the potential risk of anaphylaxis due to the unusual contexts more experienced during holidays (i.e., travels, outdoor activities and eating out). Moreover, it underlines the need for a further allergological education in these cases, in order to prepare allergic patients to avoid and manage undesired situations.

## INTRODUCTION

According to one of the definitions provided by Cambridge dictionary, holiday is “a time when someone does not go to work or school but is free to do what they want, such as travel or relax” [1]. Nevertheless, allergic patients might not agree with such a description, because of the concerns about eventual reactions due to a higher likelihood to experience unfamiliar contexts.

Such fear might turn out to be particularly true in allergic patients who experienced at least an anaphylactic reaction during their lifetime. A recent study performed on the REAACT (Recording Accidental Allergic Reactions in Children and Teenagers) cohort found a relation between vacations and higher risk for anaphylaxis (relative risk 2.3; 95% confidence interval, 1.17–4.58) [2].

Anaphylaxis is a life-threatening reaction, characterized by acute onset (minutes to several hours) and involvement of skin, mucosa or both (i.e., generalized hives, pruritus or flushing, angioedema of lips/tongue/uvula), and at least one of the following criteria: respiratory dysfunction (i.e., dyspnea, wheeze–bronchospasm, hypoxemia); persistent gastrointestinal symptoms (i.e., abdominal cramps, vomiting); decrease of blood pressure or symptoms suggesting end-organ dysfunction (i.e., syncope, incontinence). In case of exposure to a known or likely allergen, the diagnosis of anaphylaxis can be made in presence of hypotension, bronchospasm or laryngeal involvement, even in absence of the cutaneous criterion [3].

The fear of experiencing anaphylaxis in unfamiliar surroundings might affect travel planning, in terms of means of transport, journey's company and destinations chosen [4,5]. In this regard, a Danish study reported that adolescents and young adults allergic to peanuts feel uncertain or at risk when engaged in unplanned situations, considering safer the vacation with the family compared to those with friends (76% vs. 36%), local vacation compared to abroad (80% vs. 26%), and car compared to plane travel (93% vs. 40%) [4]. Holidays abroad, for example, rely with different cuisines and cultures and, for this reason, adolescents may prefer to count on parental judgment, particularly in the case of language barrier [5]. Moreover, the restrictions due to food allergies might affect also relatives’ choices. A US survey conducted on food allergic individuals highlighted that almost a half of their families restrict the number of vacations they take, with only 0.3% allowing themselves remote locations. Greater priority was given to in country destinations and closer availability of medical care, while locations least likely to be visited included Asiatic and Africans countries [6].

Another nonnegligible challenge faced by allergic patients during the holidays is the greater probability of eating out in food establishments, which is one of the main culprits for accidental exposure to allergens leading to severe anaphylaxis [2].

Unfortunately, the risk of experiencing anaphylactic reactions is not related only to food allergens, but also to the pitfalls of outdoor activities, which are more likely to be experienced during vacations. Another important category of anaphylaxis elicitors, in fact, is represented by venoms of stinging insects, such as *Hymenoptera* (wasps, bees, and ants) [7^▪▪^]. The probability to face *Hymenoptera* varies based on geographical location and season, and their stings lead to systemic reactions in adults in 3% of the cases [7^▪▪^]. In addition to the well known risks due to *Hymenoptera*, other less known arthropods may be occasionally responsible for systemic allergic reactions, such as mosquitos and horse flies [8]. Furthermore, ticks play a particular role, by representing themselves a rare cause of anaphylactic reaction [9], and at the same time by resulting strictly related to the occurrence of alfa-gal syndrome [10]. Lastly, beyond the role of allergens, particular regard should be addressed to a relevant favoring factor linked to anaphylaxis occurrence and often matched with free time, which is physical exercise. A retrospective analysis about fatal/near fatal anaphylaxis found that more severe reactions are related to exercise [11].

In light of the above, this review is aimed to summarize the potential causes of anaphylaxis in the different holiday situations, paying attention to stinging insects’ allergens and to uncommon foods not worldwide spread but more typical of particular locations. The other goal is to provide practical suggestions in order to mitigate the stress due to the challenging holiday contexts and to facilitate enjoying a really recharging free time. 

**Box 1:**
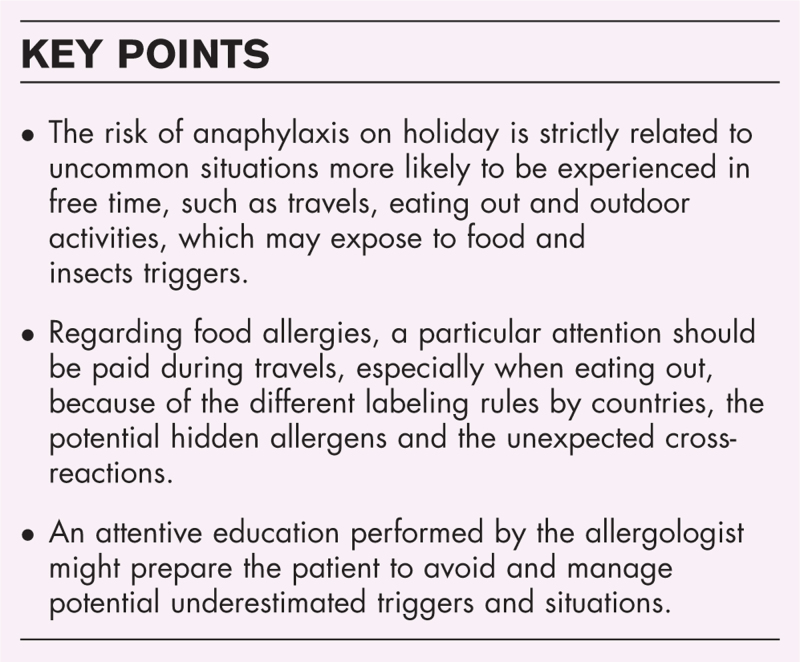
no caption available

## RISK RELATED TO ALLERGENIC FOODS DURING HOLIDAYS

According to UNWTO (United Nations World Tourism Organization), international tourism ended 2023 at 88% of prepandemic levels. The newfound desire for travel post COVID-19 pandemic, the increased air connectivity, and a recovery of Asian destinations, are expected to promote a full recovery by the end of 2024 [12]. In matter of travels, the differences in the diets characterizing various places of the world are implied in determining a significant global variation in food allergy triggers [11]. Apart from the most common allergic triggers worldwide (i.e., peanuts, tree nuts, cow's milk and crustaceans) [13], unique sources of allergens may be recognized in specific locations, and they may become particularly relevant in the perspective of potential cross-reactions or hidden allergenicity [11,14]. Valuable examples are hummus and falafel (typical of Arabian countries), noodles and natto (more typical of Asia), and couscous and bulgur, widespread African dishes made of cereal, including wheat [11,14]. Further instances are the so-called “novel foods” that, according to the European Regulation No. 2015/2283, are foods not significantly consumed in the European Union before May 15, 1997 [15]. The use of novel foods originates from the evident need to find new edible resources alternative to traditional ones, caused by the increase in world population and by the global warming [16]. They include edible insects, which are more typical of the Asiatic diets, and lupins, an emerging source of allergens in Europe [15–17]. With regard of exotic destinations, it is worthy to keep in mind the allergenic risk due to tropical fruits, especially in patients sensitized to latex [18].

Following, some examples of the potential anaphylactic triggers during a vacation trip.

### Chickpeas

In a hypothetical trip to Arabic countries, hummus and falafel would represent typical dishes, accounting chickpeas (*Cicer arietinum*) as one of the main ingredients [14]. Chickpeas major allergens include the cupins Cic a 1 (7 s vicilin) and Cic a 6 (11 s globulin). Other known allergens are Cic a 2 s albumin, Cic a 3 (lipid transfer protein – LTP) and Cic a 4 (bet v 1 like pathogen-related protein – PR10) [19,20]. Recent findings suggest a potential risk for peanut allergic patients by IgE cross-reactivity with homologous chickpea proteins, such as the Cic a 1 [20]. Cases of anaphylaxis induced by chickpea ingestion were reported, some of them related to physical exercise [21]. A clinically significant cross-reactivity was demonstrated also to lentil and pea [19].

### Lupin

Lupin (*Lupinus* spp.) is another legume broadly used as novel food and alternative source of proteins [17]. According to the Allergy Vigilance Network it is an emerging food allergen in Europe, and due to its increasing consumption it is responsible for 2.4% of food-induced anaphylaxis in French patients, a quarter of them reporting a previous history of allergy to peanut [22^▪▪^]. Such a relationship might be based on the cross reaction between storage proteins, as peanut's Ara h 1 and lupin's Lup an 1 [17], a major allergen of lupin together with Lup an 3 (LTP) and Lup a 5 (profilin) [19]. Additional cross reactivities are reported to peas and lentils [19,23]. Lupins are widely employed in prepacked food products, mainly pastries, biscuits, pasta and breads, but also in coffee, milk, ice cream and meat substitutes. Due to this variegated use, the risk of their unintentional ingestion as hidden allergens might be increased [14,22^▪▪^].

### Buckwheat

Buckwheat (*Fagopyrum* spp.) is regularly eaten in Asia as a main ingredient of noodles, bread, cakes and pancakes, especially in South Korea and Japan [11,14]. Moreover, its spreading in Western Europe is on the rise as a gluten-free alternative to grains. The major allergens of buckwheat are Fag e 1 (13 s globulin) and Fag e 2 (2 s albumin). Other known allergens are Fag e 3 (7S vicilin), Fag e 4 (antimicrobial peptide), and Fag e 5 (vicilin-like-protein) [17]. The storage proteins Fag e 2 and Fag e 5 were described as potential culprit for cross-reactivity to latex and peanuts [24]. Particularly, in a recent case report, the positivity to Fag e 2 at the component resolved diagnosis was related to a case of anaphylaxis refractory to adrenaline few minutes after the ingestion of a croissant [25].

### Soy

Soybean (*Glycine max*) is probably world's most important legume, extensively used in vegan and Asiatic cuisine, and ingested as milk or soy sauce, but also as a low-cost proteins substitute such as, miso, tempeh or tofu [17,20]. Soybean allergens with a clinical relevance are Gly m 4, a PR10 whose concentration increases with the ripening, and Gly m 5 (7 s vicilin) and Gly m 6 (11 s albumin), which cross react with peanut's Ara h 1 and Ara h 2 [20]. Soy-induced anaphylaxis usually involve adult patients and are often less severe compared to other triggers, this suggesting a relevant role for PR10 instead of storage proteins [26]. Nonetheless, Gly m 8 (2 s albumin) is described as an indicator of severe reaction [19].

In the context of soybean allergy, natto plays an exclusive role. It is a typical Japanese dish, made of soybean fermented by *Bacillus subtilis natto*. Cases of delayed anaphylaxis were related to the ingestion of natto, mostly in patients performing marine sports as surfing. The main causative allergen is poly-γ-glutamic acid (PGA) contained in natto's mucilage, with a probable sensitization mediated by jellyfish stings, whose tentacles also produce PGA [27,28].

### Edible insects

Although a recent introduction in Western countries as sustainable sources of proteins, fatty acids and minerals [15], edible insects have a long history in eastern diets, and cases of allergic reactions were longtime reported [29]. Nowadays, EFSA (European Food Safety Authority) approved only four insects as food sources (mealworm – *Tenebrio molitor*, locust – *Locusta migratoria*, lesser mealworm – *Alphitobius diaperinus*, and domestic cricket – *Acheta domesticus*) [15], but many others are widely used in Asiatic cuisines, such as bees, wasps, silkworms, bamboo caterpillars, dragonflies, and beetles [30]. The causes of reactions to edible insects may be attributable to cross-reactions due to sensitization mediated by other species, such as crustaceans, molluscs, nematodes as *Anisakis simplex*, mites or cockroaches. They all share phylogenetically preserved pan allergens, such as tropomyosin, arginine kinase, and less known ones as alpha-actin, enolase, fructose 1,6-bifhosphate aldolase, glyceraldehyde-3-phosphate dehydrogenase, apolipophorin III, larval cuticle protein and activated protein kinase receptor. Moreover, anaphylactic reactions also might be related to genuine sensitizations to chemosensory proteins, odorant-binding proteins, and hexamerins [16].

### Tropical fruits

Systemic reactions have a rate of approximately 8.7% in the global amount of the clinical presentations of fruit-induced allergy. Severe reactions occur in about 1.7%, mainly related to tropical fruits as kiwifruit, banana, mango, avocado, and durian [31^▪^]. Moreover, cases of anaphylaxis were reported as due to jackfruit, lychee and Indian jujube [32–34]. Life-threatening reactions most of the times derive from cross reactions. In the specific context of exotic fruits, but not only, a relevant instance is represented by the sensitization to latex allergens Hev b 2 (β-1,3-glucanase), Hev b 5 (acidic protein) and Hev b 6 (class 1 chitinase), responsible for latex-fruit syndrome [31^▪^]. Regarding systemic reactions attributable to pollen-food pan allergens, they are mainly caused by stable proteins as LTP, which often require precipitating co-factors to exacerbate anaphylaxis. For these reasons, patients with known sensitization to such allergen should be aware of the effect that may be provoked by some recreational activities (i.e., sport, prolonged sun exposure, alcohol and cannabis consumption), more likely to be experienced during vacations together with a lower awareness about trigger foods [35].

## RISK RELATED TO EATING OUT

Apart from travels, holiday periods might provide more eating out occasions due to larger free time, family reunions and cultural celebrations [36]. An increased risk of anaphylaxis induced by unknown nuts and peanuts was related to Halloween and Easter among Canadian children older than 6 years. Especially in the case of Halloween, children usually receive candies from unknown people that may neglect their allergies. Contrarily, Christmas is a more familiar celebration; hence, it is probably characterized by a higher vigilance on preventing the exposure to eventual allergens [36]. Regarding eating-out food establishments, 61% of vacation-related allergic reactions occurs in restaurants and hotels [2]. A study performed on the REAACT cohort reported avoiding behaviors of Irish adolescents, which were three times less likely to eat in food establishments (i.e., restaurants, cafés, and fast foods) than younger [37^▪^], which appears in contrast with findings highlighting teenage risk-taking behaviors as the delayed self-administration of epinephrine in case of anaphylaxis [38,39]. Causes of concerns dealing with eating out are scepticism on the staff's expertise and vigilance, but also the belief that asking the staff about an allergen is embarrassing [5]. In this regard, it is worthy to underline that avoiding behaviors may easily turn into the limitation of social activities, like school trips and parties, leading to negative consequences on the quality of life [3,5].

## RISK RELATED TO OUTDOOR ACTIVITIES

The greater free time on holidays is often employed to experience outdoor activities, that is, going camping, hiking, swimming, gardening or making barbecues. Such intensive exposures may lead to a greater risk for allergic patients to be stung by arthropods, as *Hymenoptera* or ticks.

Secondly, physical exercise required by some outdoor activities may act itself, in particular conditions, as a precipitating co-factor for the outbreak of anaphylactic reactions [38].

### Hymenoptera

*Hymenoptera* order include stinging insects as bees, wasps (yellowjackets, hornets, paper wasps), and ants. In case of travels, the possibility of cross reactions to venoms of related species present in different parts of the world has to be taken into account, and it may vary according to geographic locations and seasons. Climate changes and globalization are strictly related with insects’ redistribution [8]. Bees (*Apis* spp.) are established all over the world, except Antarctica, with a complete cross-reactivity among all species [7^▪▪^]. The use of domesticated bumblebees as pollinators recently leads to an increase of systemic reactions due to their stings [8]. A particular regard should be paid to Africanized bees, hybrids known as “killer bees,” notified also in the Southeast of United States. They are more dangerous compared to European bees because of their tendency to attack in swarm for long distances and to make their nests also below the ground, increasing in this way the chance to inadvertently run into unpleasant encounters [7^▪▪^]. Concerning wasps, paper wasps (*Polistes* spp.) are species very common, especially in tropical areas and in southern Italy and Spain, but less cross reactive than yellowjackets. Yellowjackets (*Vespula* spp. and *Dolichovespula* spp.) are widespread and abundant, while the highly cross-reactive hornets (*Vespa* spp.) are more noticeable in Eurasia and in northern Africa [7^▪▪^,^8^]. A certain concern arose in the last years in Europe due to the accidental importation from Asia of *Vespa velutina nigrithorax*, that spread from France to Spain, Portugal and Italy [40]. A high cross-reactivity was already related with the most common wasps’ venom. The known allergens of *Vespa velutina nigrithorax* are Vesp v 5 (homologous for >75% with antigen 5 of *Vespula* spp. and *Polistes dominula*), and Vesp v 1 (homologous for about 70% with A1-phospholipase of *Vespula* spp) [41]. Furthermore, *Vespa velutina nigrithorax* is related to a relevant number of deaths also because of some toxins in its venom leading to multiorgan dysfunction [40].

Regarding ants, among the myriad of species only few are responsible for anaphylactic reactions, as jack jumper ants (*Myrmecia pilosa*) in Australia and red fire ants (*Solenopsis invicta*), relevant in some areas of North and South America, Southeast Asia, Oceania and Caribbean islands [7^▪▪^,^8^].

### Ticks

Rare cases of anaphylaxis were reported in literature as due to tick (*Ixodes* spp. and *Amblyomma* spp.) bites [42]. By an allergological point of view, what makes ticks different from other insects is that their risk profile is not only determined by their intrinsic allergenicity, but it is worsened by their capacity to induce apparently unrelated anaphylactic reactions to food. More properly, the bite of the tick may be responsible for the sensitization to the oligosaccharide galactose-alpha 1,3-galactose (alpha-gal), a component of tick's saliva, this leading to the onset of the alpha gal syndrome, which is characterized by delayed anaphylaxis after the ingestion of nonprimate mammalian meat, dairy products and gelatine containing foods [43]. In addition, alpha gal syndrome may be responsible for systemic reactions to drugs and excipients (i.e., cetuximab, infliximab, gelatin-based colloid plasma substitutes, pancreatic enzymes, stearic acid, magnesium stearate, glycerine, and lactic acid), and gelatin-containing vaccines (measles-mumps-rubella, yellow fever, live attenuated zoster) [44].

## SUGGESTIONS FOR PREVENTING ANAPHYLAXIS ON HOLIDAYS

**FIGURE 1:**
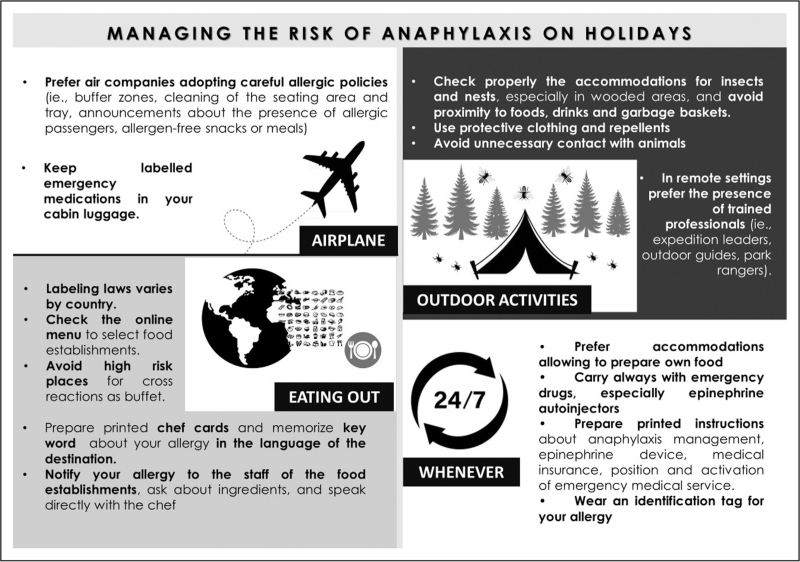
Practical suggestions to avoid and manage potential anaphylactic triggers, especially during airplane trips, outdoor activities, and eating out occasions abroad.

A careful preholiday allergological screening together with an attentive education of the patient are pivotal in the management of the patient with a previous history of anaphylaxis. Allergologist should clearly explain the anaphylactic risk according to the patient's specific vacation, and if necessary, prescribe more than one epinephrine autoinjector [45^▪▪^]. Figure 1 summarizes a list of practical and useful suggestions for allergic patients approaching to holidays, based on the different situations [7^▪▪^,^42^,^45^^▪▪^,^46^].

## CONCLUSION

The amount of free time related to holidays favors recreative situations as travels, eating out and outdoor activities. Nonetheless, such activities may result less recharging then expected for allergic patients, because of the concern of experiencing unexpected anaphylactic reactions in less familiar contexts. Regarding food allergies, different cuisines and cultures may reveals themselves as challenging: beyond the common allergic triggers, also sources less known and more typical of specific locations have to be considered because they may be responsible for cross reactions. In addition, eating out in food establishments might relate to an increasing risk due to hidden allergens, scarce vigilance or preparation of the staff, or merely because of a language barrier when abroad. Moreover, the intensive exposures during outdoor activities, as going camping or hiking, may lead to a greater risk to be stung by arthropods, increasing the probability of a systemic reaction in allergic patients. For all this reasons, a careful preholiday allergological screening together with an attentive education are pivotal in the management of the allergic patient, especially in the case of a previous history of anaphylaxis.

## Acknowledgements

*None.*

### Financial support and sponsorship

*None.*

### Conflicts of interest

*There are no conflicts of interest.*

## REFERENCES AND RECOMMENDED READING

Papers of particular interest, published within the annual period of review, have been highlighted as:

▪ of special interest
▪▪ of outstanding interest
